# Lenalidomide potentially reduced the level of cell- associated HIV RNA and improved persistent inflammation in patients with HIV-associated cryptococcal meningitis a pilot study

**DOI:** 10.3389/fcimb.2022.954814

**Published:** 2022-07-28

**Authors:** Xiang Liu, Xueling Zhu, Xiaorong Peng, Ran Tao, Zhikai Wan, Jiangjin Hui, Yongzheng Guo, Ying Hang, Biao Zhu

**Affiliations:** The Department of Infectious Diseases, State Key Laboratory for Diagnosis and Treatment of Infectious Diseases, National Clinical Research Center for Infectious Diseases, Collaborative Innovation Center for Diagnosis and Treatment of Infectious Diseases, The First Affiliated Hospital, School of Medicine, Zhejiang University, Hangzhou, China

**Keywords:** human immunodeficiency virus (HIV), HIV reservoir, inflammation, cytokines,, lenalidomide

## Abstract

**Background:**

The HIV-1 reservoir is a major barrier to curative strategies. Inflammation is an important factor for HIV-1 reservoir persistence. Lenalidomide regulates inflammatory cytokines efficiently. We examined whether lenalidomide could inhibit HIV-1 transcription and reduce systemic inflammation in people living with HIV.

**Methods:**

Lenalidomide was administered orally for 48 weeks to patients with HIV-associated cryptococcal meningitis (HIV-CM). A HIV-1 latency model was treated with or without lenalidomide ex vivo for 5 days. The primary endpoints were change in HIV reservoir markers and inflammatory cytokines in both the cohort and cell model.

**Results:**

Thirteen participants were enrolled from May 2019 to September 2020. The median change in cell-associated (CA) HIV RNA between baseline and 48 weeks was 0.81 log10 copies/million peripheral blood mononuclear cells (PBMCs). The CA HIV RNA decreased significantly in the cohort (P = 0.021). Serum tumor necrosis factor-α (TNF-α) and interleukin-6 (IL-6) gradually diminished with lenalidomide treatment until 48 weeks (P = 0.007, P = 0.014, respectively). C-reactive protein/IL-6/TNF-α and CA HIV RNA were significantly correlated (P = 0.0027, 0.0496, and 0.0346, respectively). Lenalidomide also significantly decreased HIV core P24 (P = 0.0038) and CA HIV RNA in CD8-depleted PBMCs (P = 0.0178) ex vivo. TNF-α and IL-6 were significantly reduced in the CD8-depleted PBMC supernatant (P = 0.004, P < 0.0001, respectively) while IL-10 levels increased significantly on lenalidomide compared to no-lenalidomide treatment (P < 0.0001).

**Conclusions:**

Lenalidomide was preliminarily confirmed to reduce the level of cell- associated HIV RNA and improve persistent inflammation in patients with HIV-Associated cryptococcal meningitis, which was a potential intervention for clinical use to inhibit viral transcription of the HIV-1 reservoir and reduced HIV-related inflammation in HIV-1 patients during ART.

## Introduction

Despite combination antiretroviral therapy (cART) reducing the morbidity and mortality of AIDS, HIV-1 infection remains an incurable disease (Sengupta and Siliciano, 2018; Ahlenstiel et al., 2020; Cohn et al., 2020). HIV-1 latent provirus is the major barrier to a cure, resulting in residual viremia during cART and viral rebound upon treatment cessation (Mori et al., 2020; Vansant et al., 2020). Therefore, further efforts should be expended to completely eradicate the latent provirus for a HIV-1 cure or remission.

Persistent chronic immune activation and inflammation remain the hallmark of people living with HIV (PLWH) and can also contribute to HIV-1 persistence (Palmer et al., 2018; Zicari et al., 2019; Lv et al., 2021). Under physiological conditions, HIV-1 latent cells undergo slow turnover but can divide rapidly upon stimulation by inflammatory cytokines (Palmer et al., 2018). Hence, some immunomodulators are novel therapeutic opportunities for combating this systemic immune activation/inflammation and reducing the HIV-1 reservoir.

Lenalidomide is a widely used, effective, and safe anti-tumor necrosis factor-α (TNF-α) agent (Li et al., 2018; Salles et al., 2020) that exhibits more effective immunomodulation with fewer adverse effects compared thalidomide on clinical application (Raza et al., 2017). Under different disease conditions, lenalidomide inhibited the production of proinflammatory cytokines, including interleukin-1 (IL-1), IL-6, IL-12, and TNF-α and enhanced the anti-inflammatory cytokine IL-10 (Kotla et al., 2009; Martiniani et al., 2012; López-Relaño et al., 2018). IL-6, and TNF-α can induce HIV-1 reservoir transcription, contributing to HIV persistence (Saleh et al., 2011; Vandergeeten et al., 2012), while IL-10 inhibits HIV-1 provirus activation to maintain HIV latency (Arias et al., 2010; Vandergeeten et al., 2012). Therefore, we postulated that lenalidomide might affect HIV-1 reservoir replication and reactivation by regulating inflammatory cytokine secretion.

Here, we conducted a prospective cohort study to explore the potential of lenalidomide as an immunomodulatory agent of HIV-1 patients. The initial goal of this study was to assess whether lenalidomide improves the chronic intracranial inflammatory injury of HIV-CM through 24-week treatment. Somewhat by accident, we found that lenalidomide not only improved persistent central inflammatory injury but also inhibited pro-inflammatory cytokine secretion and reduced CA HIV-RNA levels, which was not reported previously. We decided to extend the study period to 48 weeks. The inhibition of intracellular P24 expression by lenalidomide was observed in primary cells. Therefore, we preliminarily confirmed the potential of lenalidomide for reducing chronic inflammation and inhibiting HIV-1 transcription in HIV-1 patients in a clinical trial.

## Methods

### Trial population and design

This study enrolled PLWH on ART with cryptococcal meningitis (see **Supplementary Material** for the detail of Inclusion Criteria and Exclusion Criteria). These participants were successful induction therapy for AIDS-related cryptococcal meningitis and met the following criteria:a. Complete 4 weeks of induction and 8 weeks of consolidation treatment b. Two negative cerebrospinal fluid cultures for Cryptococcus. But they remained chronic inflammatory injury in the central nervous system after standard antifungal therapy. A positive diagnosis of chronically inflammatory injure in the central nervous system is cerebrospinal fluid (CSF) protein is higher than 0.45g/L or CSF white blood cell (WBC) countis greater than 8/uLor cranial radiological examination revealed abnormal signal lesions such as inflammation, edema and so on. The treatment of cryptococcal meningitis was provided in **Supplementary Table 1**. Patients with other opportunistic infections and co-morbidities were excluded. The study was approved by The First Affiliated Hospital, Zhejiang University School of Medicine (FAHZU) ethics committee and the participants provided informed consent. This study is registered with the China Clinical Trial Registry website (https://www.chictr.org/cn/, No. ChiCTR1900023184).

Lenalidomide capsules (25 mg, Celgene International Sarl, Switzerland) were administered orally on days 1–21 of a 28-day cycle for 48 weeks with continuous cART. Participants were followed-up at seven points: baseline (BL), 4 weeks (4W), 8W, 12W, 24W, 36W, and 48W. Longitudinal clinical and laboratory data were collected and included CD4^+^ T cell counts, T cell activation markers, C-reactive protein (CRP), D-dimer, serum cytokine levels, plasma viral loads, and HIV-1 DNA and cell-associated (CA) HIV-1 RNA of PBMCs. In addition, the demographic data were also obtained. The CSF sample was collected for routine CSF parameters during the first 24 weeks. Peripheral blood was collected in acid citrate dextrose tubes to obtain the PBMCs, which were isolated with SepMate (STEMCELL Technologies, Vancouver, Canada) and cryopreserved. Frozen PBMC aliquots were stored in temperature-monitored liquid N_2_ vapor-phase freezers.

### Experimental design

As CD4^+^ T cells and monocytes/macrophages are the major HIV-1 latent reservoirs and monocytes/macrophages are significant mediators of inflammation, we specifically depleted CD8^+^ T cells from the PBMCs to construct a primary HIV-1 latency model system (Kumar et al., 2014; Shapouri-Moghaddam et al., 2018). Five cART-treated aviremic PLWH were recruited at FAHZU and they provided written informed consent. They had suppressed viral loads on cART (<20 copies/mL) for >3 years and normal CD4^+^ T cell counts (>350 cells/mm^3^) for at least 6 months. PBMCs were isolated from these donors using SepMate(STEMCELL Technologies,Canada). CD8^+^ T cells were depleted from the PBMCs with CD8 MicroBeads (Miltenyi Biotec, Germany). The CD8-depleted PBMCs were cultured in complete RPMI 1640 medium supplemented with 10% fetal bovine serum and 1% penicillin-streptomycin and stimulated with 100 ng/mL lipopolysaccharide (LPS, Sigma-Aldrich,USA) in the presence or absence of 1 μM lenalidomide (MCE,USA) for 5 days. The negative control was only incubated with supplemented complete medium and dimethyl sulfoxide (DMSO). Then, intracellular P24Gag and CA HIV-1 RNA in the cells were measured. The cytokine levels were detected in the CD8-depleted PBMC supernatant.

### Measurement of HIV-1 RNA, total HIV-1 DNA, CA-HIV-1 RNA, and HIV-1 Core antigen

HIV-1 RNA was detected using a Cobas AmpliPrep/Cobas TaqMan RT−qPCR Test (Roche, USA) (lower limit of quantification = 20 copies/mL). Total cellular DNA was extracted from the PBMCs using a DNA isolation kit (Qiagen, USA) and HIV-1 DNA was quantified by a HIV quantitative detection kit (Supbio, Guangzhou, China). Cellular RNA was extracted from the PBMCs and the CA HIV RNA was measured by RT-PCR.

The PBMCs were fixed and permeabilized with a BD Cytofix/Cytoperm Kit (BD Biosciences,USA). Then, intracellular HIV-1 P24Gag staining was performed with HIV-1 core antigen–FITC (HIV-1 proteins 55, 39, 33, and 24 kD of core antigen, Beckman Coulter, USA) and examined by flow cytometry.

### Measurement of immune activation biomarkers

We measured CD4^+^ and CD8^+^ T cell activation at BL, 4W, 12W, and 24W. The PBMCs were washed and stained with Fixable Viability-R763-APCA750, anti-CD3-NUV750, anti-CD4-B525-FITC, anti-CD8-V763, anti-CD38-Y585-PE, and HLA-DR-Y763-PC7 (all from BD Pharmingen,USA). The expression of CD38^+^ and HLA-DR^+^ on CD4^+^ and CD8^+^ T cells was measured with a CytoFLEX cell analyzer (Beckman Coulter). Data were analyzed using the FlowJo V10 analysis platform. CD4^+^ T cell counts were measured by the BD Multitest IMK Kit on flow cytometry.

### Cytokine measurement

The cytokines IL-2, IL-4, IL-6, IL-10, TNF-α, interferon (IFN)-γ, and IL-17A were determined using a cytometric bead array flow cytometric assay (cat. no. 560484, Human Th1/Th2/Th17 CytokineKit, BD Biosciences). Standard curves were constructed and quantitative calculations were performed using FCAP Array Software (BD Biosciences).

### Statistical analysis

The longitudinal dynamics of the HIV-1 reservoir and inflammatory biomarkers were modeled using linear regression. Correlations of the biomarkers of inflammatory and the HIV-1 reservoir were determined by Spearman tests. The experimental data are expressed as the median (interquartile range [IQR]). The differences between groups were statistically analyzed with the Student t-test or ordinary one-way analysis of variance. The statistical analyses were performed with IBM SPSS Statistics 22(Chicago, USA) and Prism 8 (GraphPad,USA). P < 0.05 was considered statistically significant.

## Results

### Participants’ characteristics at baseline

From May 2019 to December 2021, we enrolled 13 patients in the case series and completed the follow-up according to the study protocol. Prior to this study, the participants had received standard antifungal therapy including induction and consolidation treatment, and eight had a ventriculoperitoneal shunt inserted to control CSF pressure and hydrocephalus(**Supplementary Table 1**).

All participants were male and Han Chinese and the median age was 32 years (IQR 30–39 years). The median CD4^+^ lymphocyte count was 184 cells/μL (IQR, 131–342 cells/μL): eight participants had 100–199 CD4^+^ T cells/μL, two had 200–350 CD4^+^ T cells/μL, and three had >350 CD4^+^ T cells/μL. Ten participants had HIV-1 RNA below 50 copies/mL and three had low-level HIV viremia (200–500 copies/mL) at study entry. **Table 1** shows the individual participants’ characteristics and cART regimen.

**Table 1:** Participants’ Characteristics at Baseline .

Characteristic	Number or median	Percentage or IQR
Age (year), median (IQR)	32	(30–39)
Sex, n (% of cohort)		
Male	13	100%
Time since HIV diagnosis (months), median (IQR)	18	(13-24)
Time on cART (months), median (IQR)	16	(12-21)
cART regimen, n (% of cohort)		
TDF+3TC+EFV	2	15
TDF+3TC+DTG	9	69
TDF+ 3TC+LPV/r	1	7.5
BIC/FTC/TAF	1	7.5
CD4 count at inclusion, n (% of cohort)		
100 to 199 CD4^+^/μl	8	62%
200 to 350 CD4+/μl	2	15%
>350 CD4+/μl	3	23%
Plasma HIV-1 RNA, n (% of cohort)		
<50 copies/mL	10	77%
50-200 copies/mL	0	0%
200-500 copies/mL	3	23

TDF, tenofovir disoproxil fumarate; 3TC, lamivudine; EFV, efavirenz; DTG, dolutegravir; LPV/r, lopinavir/ritonavir; BIC/FTC/TAF, bictegravir/emtricitabine/tenofovir alafenamide fumarate.

### Safety evaluation of lenalidomide

We analyzed the safety of lenalidomide by the adverse effects and laboratory data during follow-up in 13 participants. Two (15.3%) patients developed a mild rash that resolved spontaneously without dosing interruption. No study drug-related serious adverse events were reported, and no participant died during the study period. No patient withdrew from treatment due to unacceptable toxicity. Furthermore, our study showed that lenalidomide had a modest suppressive effect on bone marrow and a greater effect on neutrophils. We found that there was a slight decrease in white blood cells, neutrophils and platelets during the follow-up period. The lowest values of median leukocyte and platelet counts occurred at week 12(4.10 x10^9^/L IQR 2.75-4.90, P=0.002) and week 8 (173.00 x10^9^/L IQR 130.00-189.50, P<0.001)respectively, which remained within the normal range. In addition, more laboratory indicators like liver and kidney functions, blood lipids, blood urine nitrogen, total bilirubin and coagulation functions were found no obvious change during the follow-up period. And there was no significant differences in the CD4+T-cell and CD8+T-cell counts following lenalidomide therapy. (See **Supplementary Table 2** for details)

### Lenalidomide reduced the participants’ serum TNF-α and IL-6 levels

**Figure 1:**
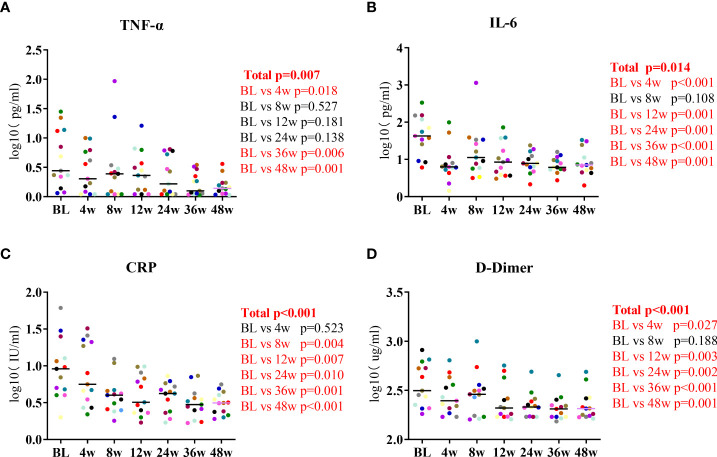
Longitudinal dynamics of **(A)** TNF-α, **(B)** IL-6, **(C)** CRP, and **(D)** D-dimer during the 48-week lenalidomide therapy. Participants are color-coded. *P*-values representing the difference between the time points are shown on the right side of the graphs.

We measured the participants’ levels of inflammatory biomarkers at the seven timepoints of lenalidomide therapy. The changes in IL-2, IL-4, IL-10, IFN-γ, and IL-17A levels were not significant at the end of lenalidomide treatment. While serum TNF-α levels decreased by a mean of 0.46 log10 pg/mL between baseline and 48W, the mean decrease in IL-6 levels in the same period was 0.72 log10 pg/mL, which were significant (P = 0.007, P = 0.014, respectively, **Figures 1A, B**). CRP decreased at 8W and subsequently remained relatively stable (P < 0.001, **Figure 1C**) and D-dimer levels gradually diminished during the 48-week treatment (P < 0.001, **Figure 1D**). Taken together, these results indicate that lenalidomide can reverse aberrant immune activation and reduce HIV-related immune responses. We also analyzed routine CSF parameters of 13 patients during the first 24 weeks. A significant decrease was observed in the levels of CSF WBC count, glucose, protein, and ALB after 24 weeks compared with the baseline (**Supplementary Table 3**). This suggests that lenalidomide ameliorated the persistent central inflammatory damage in HIV-CM patients.

### Lenalidomide attenuated the participants’ CA HIV-1 RNA levels

**Figure 2:**
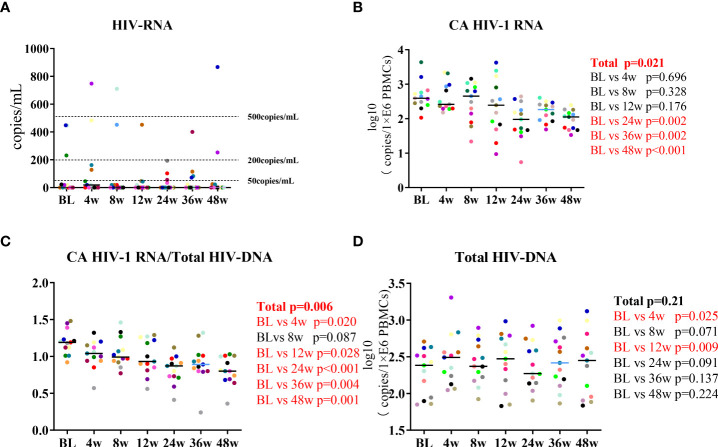
Longitudinal dynamics of **(A)** HIV-1 RNA, **(B)** CA HIV-1 RNA, **(C)** CA HIV-1 RNA/total HIV-1 DNA, and **(D)** total HIV-1 DNA during the 48-week lenalidomide therapy. Participants are color-coded. *P*-values representing the difference between the time points are shown on the right side of the graphs.

In this prospective cohort, we measured the participants’ HIV-1 RNA, total HIV-1 DNA, and CA HIV RNA. The 13 HIV-CM patients maintained HIV-1 RNA suppression during the 48 weeks, with residual viremia in three patients that remained at <500 IU/mL (**Figure 2A**). Changes in HIV-1 DNA and CA HIV-1 RNA after lenalidomide treatment were observed. The mean change in CA HIV-1 RNA between BL and 48W was 0.81 log10 copies/million PBMCs. CA HIV-1 RNA decreased during the first 24 weeks on lenalidomide (P =0.002) and also decreased significantly during lenalidomide treatment (P =0.021, **Figure 2B**). The change in CA HIV-1 RNA/total HIV DNA was statistically significant (P = 0.006, **Figure 2C**). Despite total HIV-1 DNA increasing at 4W and 12W, there was no significant reduction after the end of the lenalidomide treatment (P = 0.21, **Figure 2D**). Based on these findings, we concluded that lenalidomide blocked HIV-1 transactivation of the latent provirus in the cohort.

### Correlation between inflammatory markers and CA HIV RNA

**Figure 3:**
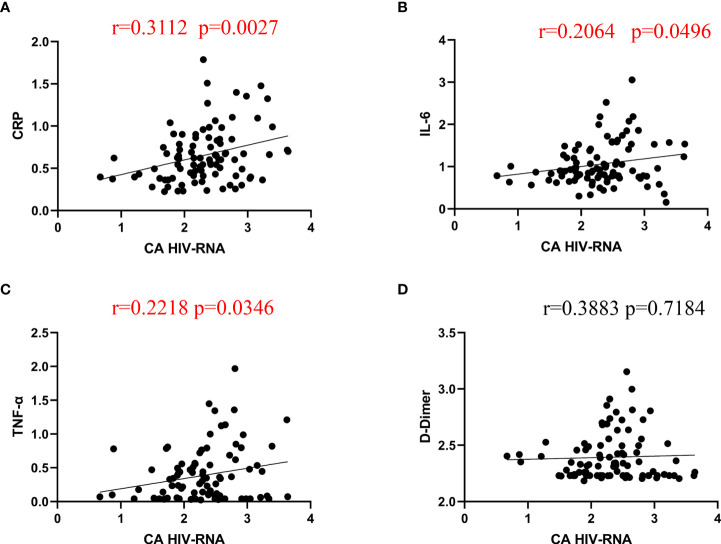
Spearman correlations between CA HIV-1 RNA and CRP, IL-6, TNF-α, and D-dimer. **(A–C)**, CRP, IL-6, and TNF-α were significantly correlated with CA HIV RNA levels after lenalidomide therapy. **(D)**, The correlation between D-dimer and CA HIV-1 RNA was not significant.

As the inflammatory biomarkers and CA HIV RNA both decreased after lenalidomide therapy, we determined whether the inflammatory biomarkers CRP, D-dimer, IL-6, and TNF-α were correlated with the reduction in CA HIV RNA. There were significant correlations between CRP, IL-6, TNF-α, and CA HIV RNA (**Figure 3**) (P = 0.0027, 0.0496, and 0.0346, respectively), while the correlation between D-dimer and CA HIV-1 RNA was not significant (P = 0.7184).

### Lenalidomide did not induce T cell activation

**Figure 4:**
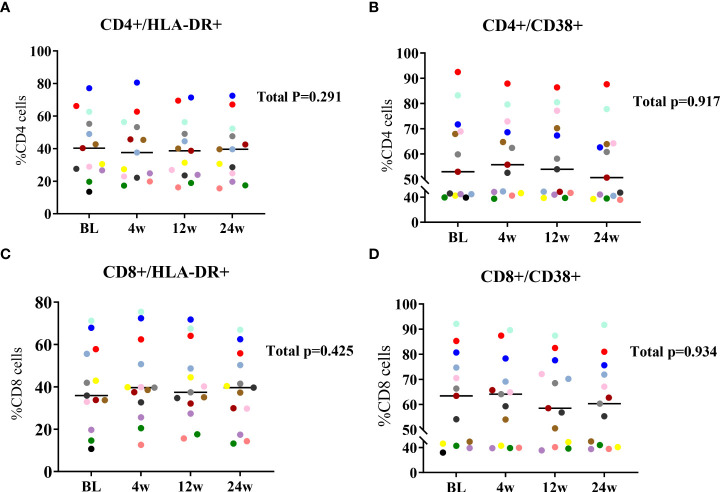
Longitudinal dynamics of **(A, B)** CD4^+^ and **(C, D)** CD8^+^ T cell activation at 24W of lenalidomide therapy. Participants are color-coded. *P*-values representing the values of the change during lenalidomide treatment are shown on the right side of the graphs.

We evaluated the effect of lenalidomide on the expression of T cell activation markers (i.e., the percentages of CD38^+^ and HLA-DR^+^) at BL, 4W, 12W, and 24W with flow cytometry. CD4^+^ T cell activation levels were relatively stable and did not change significantly during 24 weeks of lenalidomide treatment (**Figures 4A, B**). Similar to CD4^+^ T cells, CD8^+^ T cell activation did not significantly decrease or increase (**Figures 4C, D**). Our results suggest that lenalidomide did not induce T cell activation.

### Lenalidomide inhibited HIV-1 reactivation of CD8-depleted PBMCs

**Figure 5:**
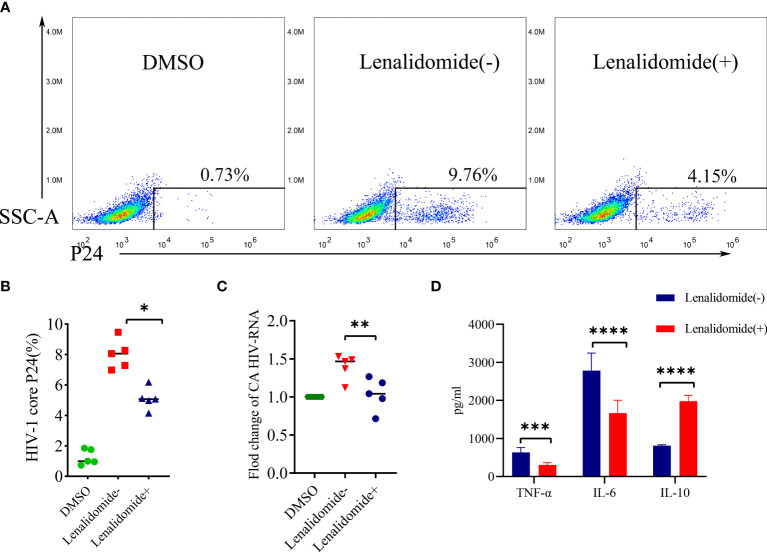
Lenalidomide inhibited HIV-1 reactivation in CD8-depleted PBMCs. CD8-depleted PBMCs were treated with TNF-α and LPS with or without lenalidomide for 5 days, or with the solvent control. **(A, B)**, HIV core antigen was determined by flow cytometry in the PBMCs. **(C)**, CA HIV RNA of CD8-depleted PBMCs was measured by RT-PCR. **(D)**, Cytokine levels in the supernatants were determined by flow cytometry. *P < 0.05, **P < 0.01, *** *P*< 0.001, ****P < 0.0001.

We validated the efficacy of lenalidomide in inhibiting HIV-1 reactivation ex vivo. CD8-depleted PBMCs with HIV-1 latency were stimulated with LPS in the presence or absence of lenalidomide for 5 days. As showed in **Figure 5A**, the HIV-1 core P24 from one patient in lenalidomide (+) group decreased from 9.76% to 4.15% as compared to the lenalidomide (-) group, while that of the DMSO control was 0.73%. Lenalidomide significantly reduced the percentage of HIV-1 core P24 in five samples (P = 0.0101, **Figure 5B**). The mean CA HIV-1 RNA number was 0.49-fold lower in the lenalidomide treatment group compared to the lenalidomide (-) group and was statistically significant (P = 0.0018, **Figure 5C**). Together, these data from the primary HIV-1 latency model system demonstrate that lenalidomide is a potent inhibitor of viral reactivation of HIV-1 latent cells.

### Lenalidomide regulated TNF-α, IL-6, and IL-10 levels in CD8-depleted PBMC supernatant

We also investigated whether lenalidomide could regulate cytokine secretion in CD8-depleted PBMC supernatant effectively. Supernatant cytokine levels were determined using a cytometric bead array kit. TNF-α and IL-6 levels in the supernatant were reduced, while IL-10 was increased in the lenalidomide-treated group compared to the lenalidomide (-) group. TNF-α and IL-6 decreased significantly after lenalidomide treatment (P = 0.004, P < 0.0001, respectively), while IL-10 increased significantly (P < 0.0001) (**Figure 5D**).

## Discussion

Our findings provide evidence that lenalidomide is safe, well-tolerated, and ameliorated the persistent central inflammatory damage in HIV-associated cryptococcal meningitis patients. Moreover, lenalidomide induces a sustained decrease in plasma IL-6, TNF-α, CRP, D-dimer, and CA-HIV-1 RNA from PBMCs in patients with HIV-associated cryptococcal meningitis. Further study indicated that lenalidomide suppressed CA HIV-RNA by inhibiting aberrant immune activation.

More recently, inhibiting HIV-1 latent provirus replication is an increasingly attractive approach for a HIV-1 functional cure strategy. Here, we observed that lenalidomide and continuous cART achieved a mean change of 0.81 log10 copies/million PBMCs in the CA HIV-1 RNA of HIV-CM patients with >1 year HIV-1 RNA suppression. Moreover, lenalidomide reduced CA HIV-1 RNA by 0.45-fold in CD8-depleted PBMCs. Similarly, didehydro-cortistatin A (dCA) reduced the mean viral RNA level per 10^5^ CD4^+^ T cells from BLT mouse tissues by 3.8-fold (Kessing et al., 2017). Mori et al. demonstrated that spironolactone treatment achieved an average of 1.4 log (27.6-fold) per million cell reduction of HIV-1 reactivation (Mori et al., 2020). CA HIV-1 RNA decreased in both the spleen (17.7-fold) and bone marrow (4.4-fold) in HIV-1-infected mice treated with IFN-α/β receptor (IFNAR) antibody (Cheng et al., 2017). These approaches appear more efficient than lenalidomide treatment for inhibiting HIV-1 transcription from latent reservoirs. Nevertheless, considering that our results were obtained from a cohort study, lenalidomide is a potent inhibitor of viral reactivation from HIV-1 reservoirs. However, lenalidomide treatment in our study did not alter the total HIV-1 DNA and the same result was reported for dCA-treated rhesus macaques chronically infected with simian immunodeficiency virus (Mediouni et al., 2019), indicating that the reduction of CA HIV-1 RNA results from lenalidomide activity on HIV-1 transcription and not the loss of proviruses.

Chronic immune system activation is one of the strongest predictors of HIV infection outcome, which is the key mediator of non-AIDS comorbidities during ART (Zicari et al., 2019; Lv et al., 2021). Specific cytokines, including IL-6 and TNF-α, have been implicated as key factors in chronic inflammation and HIV progression (Tenorio et al., 2014 ; Kamat et al., 2012). As we expected, IL-6, TNF-α, CRP, and D-dimer levels were all reduced after lenalidomide treatment, which is consistent with previous findings that lenalidomide attenuated IL-6 and TNF-α secretion in a pulmonary fibrosis model (Li et al., 2022) and mantle cell lymphoma (Ma et al., 2017). Furthermore, the Spearman correlograms revealed correlations between CA HIV RNA and IL-6, TNF-α, and CRP, indicating that regulating specific cytokines contributed to suppressing HIV-1 activation and transcription from latent reservoirs. As JAK1/2 inhibitors, ruxolitinib and tofacitinib reduced systemic inflammation and inhibited HIV-1 replication and reactivation in a macaque model (Gavegnano et al., 2014). Moreover, anti-IFNAR antibodies counteracted the excessive immune activation by targeting the chronic IFN signaling cascade on target cells, and blocking type I IFN signaling reduced HIV-1 reservoirs (Cheng et al., 2017; Zhen et al., 2017). IFNR blockade significantly reduced PD-1, HLA-DR, and CD38 expression levels on CD4^+^ and CD8^+^ T cells, indicating reduced T cell activation. However, we observed that the expression of activation markers (CD38 and HLA-DR) on T cells was unchanged during lenalidomide therapy. A possible explanation for the difference is that lenalidomide and anti-INFAR antibody attenuate inflammation through different mechanisms. At the very least, lenalidomide did not induce T cell activation. Many small-molecule compounds have failed to eliminate HIV latent proviruses or prevent latent provirus rebound due to global activation in human T cells, which results in adverse effects and toxicity by affecting cellular homeostasis (Bullen et al., 2014; Laird et al., 2015).

It was confirmed that lenalidomide is a TNF-α antagonist that blocks the TNF–TNF receptor (TNFR) pathway in previous study (Morabito et al., 2019; Blair, 2020). TNF–TNFR signaling contributes to the establishment and maintenance of HIV-1 latency (Herbein and Khan, 2008; Pasquereau et al., 2017). TNF–TNFR pathway blockade could be useful for modulating viral reservoirs, as HIV infection is characterized by immune activation and inflammation (Pasquereau et al., 2017). Thalidomide reduces serum TNF levels and controls the viral load (Marriott et al., 1997; Gallitano et al., 2016). In our study, the ability of lenalidomide to inhibit TNF-α and IL-6 and decrease HIV-1 replication suggested that blocking the TNF–TNFR signaling pathway could be the main mechanism of lenalidomide suppression of viral replication in HIV-1 latent reservoirs. Therefore, we preliminarily confirmed that lenalidomide is as a TNF-α antagonist in suppressing HIV-1 reservoir activation and replication.

This study has several limitations. First, the biggest limitation is lacking a control group in our study. A single-arm clinical study does have limitations for evaluating the effects of lenalidomide. As an exploratory study, we ultimately found that lenalidomide was the drug with the potential to inhibit HIV-1 transcription and reduce HIV-related inflammation. In the next phase, we plan to conduct a randomized clinical trial in population with fully suppressed plasma viremia while receiving ART to validate the effect of lenalidomide. Second, the use of total HIV DNA and CA HIV RNA to measure the reservoir has its limitations although it aligns with previous work in this area. It does not discriminate between actively or latently infected cells, nor if the detected proviruses are replication competent or defective. Our cohort did not have a normal immune system and the results may not be generalizable. Lastly, we were unable to elucidate the mechanism of lenalidomide on the HIV reservoir. Further studies are needed to investigate whether lenalidomide inhibits HIV-1 transcription *via* the TNF–TNFR signaling pathway.

To our knowledge, this is the first prospective study with clinical data and biologic specimens collected specifically to address the role of lenalidomide on the HIV reservoir. Lenalidomide is widely used because of its immunomodulatory and anti-inflammatory effects. Our study demonstrated that lenalidomide treatment was a potential intervention for clinical use to reduce inflammation and inhibit viral replication in PLWH during ART.

## Data availability statement

The original contributions presented in the study are included in the article/**Supplementary Material**. Further inquiries can be directed to the corresponding author.

## Ethics Statement

The studies involving human participants were reviewed and approved by The First Affiliated Hospital, Zhejiang University School of Medicine (FAHZU) ethics committee. The patients/participants provided their written informed consent to participate in this study.

## Author contributions

BZ, XP, and RT developed the concept and designed the study. XP, RT, XL, and YH provided study materials and participants. XL and XZ completed the ex vivo benchtop study. XL, XZ, ZW, JH, and YG verified the data and performed the statistical analyses. XL, XP, and XZ wrote the initial draft of the manuscript. All authors provided critical comments and editing, contributed to the data interpretation, and reviewed the analyses of this manuscript and approved its final version.

## Funding

This work was supported by the National Key R&D Program of China (No.2021YFC2301900- 2021YFC2301901) and the National Special Research Program for Important Infectious Diseases [grant number 2017ZX10202102].

## Acknowledgments

The authors thank the participants of this study, and the staff of the Infectious Ward of The First Affiliated Hospital of Zhejiang University, and the State Key Laboratory for Diagnosis and Treatment of Infectious Diseases.

## Conflict of Interest

The handling editor BS declared a past co-authorship with the author BZ. The authors declare that the research was conducted in the absence of any commercial or financial relationships that could be construed as a potential conflict of interest.

## Publisher’s note

All claims expressed in this article are solely those of the authors and do not necessarily represent those of their affiliated organizations, or those of the publisher, the editors and the reviewers. Any product that may be evaluated in this article, or claim that may be made by its manufacturer, is not guaranteed or endorsed by the publisher.
