# Supplementary material for: Lenalidomide potentially reduced the level of cell- associated HIV RNA and improved persistent inflammation in patients with HIV-associated cryptococcal meningitis a pilot study
Source: Front Cell Infect Microbiol. 2022 Jul 28;12:954814. doi: 10.3389/fcimb.2022.954814 (PMC9369255; doi:10.3389/fcimb.2022.954814)
Supplement: Supplementary file 1 [file DataSheet_1.docx]

**Supplementary Material**

**Definition of primary HIV infection**

1.High-risk exposure within previous 6 weeks

2.Detectable virus in plasma (p24 Ag and/or HIV-RNA) and/or evolving anti-HIV antibody reactivity (negative or indeterminate to positive)

3.With or without clinical symptoms

4. Everyone with detectable HIV-VL and negative or indeterminate serology must receive confirmation of anti-HIV antibody seroconversion in follow-up testing. The interval of testing is one week.

**Inclusion Criteria**

1. HIV-1 infected individuals

2. Cryptococcal disease diagnosed pre-ART by positive culture or typical clinical features plus positive India ink staining or antigen detection

3. Successful induction therapy for AIDS-related cryptococcal meningitis: a.Complete 4 weeks of induction and 8 weeks of consolidation treatment b. Two cerebrospinal fluid cryptococcal cultures were negative

4. Taking antiretroviral therapy >12 months

5. plasma HIV-1 RNA below 500 copies per milliliter

6. Patients with chronic inflammation of the central system (presence of one or more of the following)

1. CSF protein is higher than 0.45g/L

2. CSF nucleated cell count is greater than 8/uL

3. Cranial radiological examination revealed abnormal signal lesions such as inflammation, edema and so on

7. Patients are willing to give informed consent

**Exclusion Criteria**

1. Pregnant or breast-feeding

2. Patients with poor treatment compliance

3. Patients who have been treated with immunosuppressants or other immunomodulators or cytotoxic drugs within 6 months before screening

4. Patients with severe underlying diseases of the heart, brain, liver and kidney

5. Absolute neutrophil count at 1000 cells per μL or less, platelet count less than 75000/ul

6. A known hypersensitivity or contraindication to lenalidomide

7. Participated in other clinical trials within 3 months

8. Patients with severe mental illness
