# Supplementary material for: Lenalidomide potentially reduced the level of cell- associated HIV RNA and improved persistent inflammation in patients with HIV-associated cryptococcal meningitis a pilot study
Source: Front Cell Infect Microbiol. 2022 Jul 28;12:954814. doi: 10.3389/fcimb.2022.954814 (PMC9369255; doi:10.3389/fcimb.2022.954814)
Supplement: Supplementary file 2 [file Table_1.docx]

**Table S1 The treatment of cryptococcal meningitis**

| ID | Time CM diagnosis (months) | Induction  period  regimen | Consolidation period regimen | Ventriculoperitoneal shunt inserted(yes 1,no 2) | secondary prophylaxis of CM(yes 1,no 2) |
| --- | --- | --- | --- | --- | --- |
| 001 | 12.1 | AmB+flucytosine | fluconazole | 1 | 1 |
| 002 | 46.9 | AmB+flucytosine | fluconazole | 1 | 0 |
| 003 | 12.2 | AmB+flucytosine | fluconazole | 1 | 1 |
| 004 | 50.0 | AmB+flucytosine | fluconazole | 0 | 0 |
| 005 | 21.6 | AmB+flucytosine | voriconazole | 1 | 0 |
| 006 | 10.9 | AmB+flucytosine | fluconazole | 0 | 1 |
| 007 | 18.8 | AmB+flucytosine | fluconazole | 0 | 0 |
| 008 | 11.7 | AmB+flucytosine | fluconazole | 1 | 1 |
| 009 | 20 | AmB+flucytosine | fluconazole | 1 | 0 |
| 010 | 24.7 | AmB+flucytosine | fluconazole | 1 | 0 |
| 011 | 16 | AmB+flucytosine | fluconazole | 0 | 1 |
| 012 | 44.2 | AmB+flucytosine | fluconazole | 1 | 0 |
| 013 | 25.6 | AmB+flucytosine | fluconazole | 0 | 0 |

CM: Cryptococcal meningitis; AmB: Amphotericin B
