# Supplementary material for: Lenalidomide potentially reduced the level of cell- associated HIV RNA and improved persistent inflammation in patients with HIV-associated cryptococcal meningitis a pilot study
Source: Front Cell Infect Microbiol. 2022 Jul 28;12:954814. doi: 10.3389/fcimb.2022.954814 (PMC9369255; doi:10.3389/fcimb.2022.954814)
Supplement: Supplementary file 3 [file Table_2.docx]

**Table S2 Safety analysis of lenalidomide**

| Time | Baseline | 48 Weeks | *P* value |
| --- | --- | --- | --- |
| Routine blood test |  |  |  |
| HB (g/l) | 133.50 (129.50-139.25) | 134.00 (131.00-145.00) | 0.683 |
| WBC (10E9/l) | 6.80 (5.48-8.33） | 4.17（3.30-4.60） | **0.041** |
| N (10E9/l) | 3.75(2.60-4.85) | 1.28（1.10-2.00） | **0.016** |
| PLT (10E9/l) | 259.50 (207.25-303.75) | 241.00 (182.00-216.00) | 0.057 |
| E (10E9/l) | 0.20 (0.14-0.36） | 0.19（0.06-0.31） | 0.056 |
| Biochemistry |  |  |  |
| ALT (U/l) | 19.00（12.00-28.25） | 19.00(18.00-30.00) | 0.260 |
| AST(U/l) | 15.00(9.50-20.25) | 17.00(14.00-22.00) | 0.285 |
| TB (umol/l） | 5.80(3.65-7.40) | 6.75 (4.68-10.28) | **0.022** |
| ALB (g/l) | 41.55 (39.55-42.58) | 41.70(38.70-43.00) | 0.505 |
| Cr (umol/l） | 84.50 (79.75-99.25) | 90.00 (85.00-97.00) | 0.689 |
| BUN (umol/l） | 4.60 (3.38-5.04) | 5.25(4.07-5.76) | **0.013** |
| TG (mmol/l） | 1.83 (1.29-3.52) | 2.27 (1.43-2.74) | 0.824 |
| TC (mmol/l） | 3.60 (3.19-4.52) | 4.02 (3.46-4.22) | 0.790 |
| CRP (mg/ml） | 3.40 (1.50-9.37） | 3.20(1.30-5.20) | 0.398 |
| Coagulation function |  |  |  |
| PT (s) | 11.45 (10.98-1.75) | 11.400 (10.80-11.70) | 0.440 |
| CD4+T cell (cells/ul) | 151.50 (117.00-342.25) | 202.00 (137.00-345.00) | 0.091 |
| CD8+T cell (cells/ul) | 1038.00 (645.75-1459.75) | 1198.00 (896.00-1296.00) | 0.594 |

Bold font indicates p < 0.05; HB: hemoglobin; WBC: white blood cells; N: neutrophils; PLT: platelet; E: eosinophil; ATL: alanine aminotransferase; AST: aspartate aminotransferase; TB: total bilirubin; ALB: albumin; Cr: creatinine; BUN: blood urea nitrogen; TG: triglyceride; TC: total cholesterol; CRP: c-reactive protein; PT: prothrombin time;
