# Supplementary material for: Lenalidomide potentially reduced the level of cell- associated HIV RNA and improved persistent inflammation in patients with HIV-associated cryptococcal meningitis a pilot study
Source: Front Cell Infect Microbiol. 2022 Jul 28;12:954814. doi: 10.3389/fcimb.2022.954814 (PMC9369255; doi:10.3389/fcimb.2022.954814)
Supplement: Supplementary file 4 [file Table_3.docx]

**Table S3 Change in routine CSF parameters from baseline to after 24 weeks of treatment**

| Time | Baseline | 24 Weeks | *P* value |
| --- | --- | --- | --- |
| Open pressure | 150.00(116.25-217.50) | 150.00 (120.00-175.00) | 0.533 |
| CSF WBC | 35.00(4.50-90.00) | 10.00(2.00-14.00) | **0.009** |
| CSF chlorine | 123.50 (118.75-127.25) | 126.00 (121.00-128.00) | 0.155 |
| CSF glucose | 2.60 (2.10-2.90) | 2.70(2.50-2.80) | **0.044** |
| CSF protein | 1.39 (0.74-3.23) | 0.75 (0.52-1.08) | **0.003** |
| CSF ALB | 79.20 (48.40-149.75) | 42.35 (32.68-54.40) | **0.017** |
| CSF IgG | 13.10 (7.26-160.50) | 11.20 (6.43-36.00) | 0.263 |

Bold font indicates p < 0.05; HB: hemoglobin; CSF: cerebrospinal fluid；WBC: white blood cells；ALB: albumin; IgG: Immunoglobulin G
